# A philosophical treatise on information, consciousness, and the architecture of perceived reality

**DOI:** 10.3389/fpsyg.2026.1835944

**Published:** 2026-06-09

**Authors:** Phillip A. Martin

**Affiliations:** Saint Leo University, St. Leo, FL, United States

**Keywords:** consciousness, evolutionary neuroscience, golden ratio, information-theoretic substrate, microtubules, philosophy of physics, predictive processing, topological quantum field theory

## Abstract

This treatise presents a provisional interdisciplinary framework that synthesizes established findings from physics, neuroscience, and information theory to address questions that orthodox disciplines have addressed only in isolation. The argument unfolds in layers, each building on the last, and asks the reader to hold conclusions provisionally until the full architecture is visible. The central proposal is that what we experience as reality may be understood as a constructed model generated by a biological prediction engine operating on delayed information, and that the substrate of that information may be described as a pervasive field of topological information states whose defining characteristic is intrinsic expansion. This is offered as a candidate mechanism rather than an established finding. Existing findings in physics and information theory suggest directions consistent with this interpretation, though complete formalization remains to be developed. To move beyond metaphor, we present: (i) a reaction–diffusion formalism in which the golden ratio *φ* acts as a dynamical attractor, (ii) a layered topological field construction that permits anyon-like behavior at physiological temperatures, (iii) a SHEPHERD filtration sequence connecting field dynamics to perception, and (iv) four quantitative predictions that are in principle falsifiable. All speculative extensions are clearly distinguished from empirically grounded claims. The framework is designed to unfold as it progresses, with each section building on the last and each conclusion opening into the next question. The reader who stops at any point will have encountered something complete. The reader who continues will find it deepening.

## 1 Preface: The view from the void

I am aware of how this work appears on a curriculum vitae. I am not a theoretical physicist. I am not a neurosurgeon; I am an unaffiliated independent researcher. By the standards of the academy, I am a case manager with a double-major bachelor’s degree — and this work emerges from outside the institutional frameworks that typically govern contributions to these fields.

What is delineated herein is not a creation, nor even wholly a synthesis of my own making. It is a group of observations that appear to have intrinsic value to our species when proper collaboration and consideration take place. The author requests that the reader engage with the work in its entirety before evaluating any individual claim in isolation.

A theory is a specific type of creation, one that does indeed appear rather unique to our species. An observation, however, is a response to environmental stimuli. The adaptive responses of certain flora, fauna, and even protozoa suggest that observation and adaptation are critical to life. On the same note, many theoretical models of our species’ mutual interests have resulted in the detriment of our species, as well as many others.

As a person with complete aphantasia, I have no visual representation associated with neural processing. This, as with any neurodivergence, has come with its own set of adaptations given the benefits and challenges it entails. In my youth, I found peace in the cognitive scaffolding of Giordano Bruno. Images were replaced by relationships; relationships that, over time, appeared suggestive of a certain sort of “meaning.”

What follows in this treatise is not a set of conclusions reached through textbook study, but an account of observational patterns and the structural relationships they appear to encode. These relationships and correspondences appear to represent a single, coherent geometry.

Because this mode of cognition does not organize experience around visual objects, it instead organizes experience around processes and relationships. The structural pattern this framework designates as the braid emerged from sustained attention to relational rather than representational features of experience—what remains when object-based organization is not available as a primary cognitive tool.

On the Provisional Nature of This Work: Several sections present mathematical formalisms that are candidate representations rather than completed derivations. These are offered as scaffolds for collaborative development, not as established results. Where claims extend beyond current empirical verification, this is explicitly noted. The framework’s value lies not in its current completeness but in its capacity to generate testable questions and invite interdisciplinary engagement.

"Peace if possible, truth at all costs" — Martin Luther

## 2 Philosophical introduction: what this work asks of you

The argument that follows is not presented as settled science. It is offered as a working framework—a theoretical synthesis that asks the reader to hold its conclusions provisionally, to follow the logic before evaluating the premises, and to resist the impulse to categorize it before seeing what it does.

The framework draws on physics, neuroscience, and the philosophy of mind. It does not belong exclusively to any of these disciplines, so it will, at times, ask readers trained in each to hold their disciplinary commitments lightly. The physicist will encounter claims that have not yet been formally derived. The neuroscientist will encounter philosophical territory. The philosopher will encounter physics presented in the first person by a theorist, not in the third person of a textbook. This is not carelessness. It is a necessary condition for interdisciplinary study at the edge of what existing disciplines have addressed individually.

The framework emerged from sustained observational work rather than from deductive design. It does not ask for agreement; it asks for serious engagement. If the framework is wrong, demonstrating that will require engaging with topological information theory, quantum biology, consciousness science, and the comparative cognition literature. Together, that engagement is likely to be productive regardless of its verdict.

This is an invitation to a particular kind of thinking: patient, provisional, and willing to follow implications where they lead before deciding whether to accept the premises that produced them. The work requests rather than demands; it asks only that the reader engage with the full architecture before rendering a verdict on any part of it.

## 3 Quintessence and the phi vector: the substance and its nature

There is a number that elaborates itself.

*φ*—the golden ratio, approximately 1.618—satisfies a relation unique among all numbers: φ^2^ = φ + 1. It is self-similar under its own operation. Divide a line in the golden proportion, and the ratio of the whole to the larger part equals the ratio of the larger part to the smaller. The pattern reproduces at every scale. It does not simplify toward equilibrium. It elaborates.

This property is not confined to mathematics. It appears in biological systems with a frequency and independence that demand a deeper question. The spiral arrangements of seeds in sunflowers and pinecones—phyllotaxis—follow Fibonacci sequences that converge on *φ* across unrelated plant lineages (Livio, 2002). The mechanistic explanation—packing efficiency and inhibition zone dynamics in meristematic tissue—is well established (Douady and Couder, 1992). Vascular branching ratios in mammalian cardiovascular systems approximate *φ* (Zamir, 1999). The cochlea of the human inner ear is described as a logarithmic spiral whose expansion ratio approaches the golden proportion (Manoussaki et al., 2008).

These mechanistic accounts are accurate. They described how φ emerges in each system. What they do not address is the prior question: why do the universe’s optimization functions—packing efficiency, minimum energy expenditure, acoustic resonance—converge on this particular ratio across such disparate domains? The ‘how’ is documented. The ‘why’ points to something the mechanisms alone cannot reach.

The philosophical traditions that asked this question arrived at a consistent answer, even without the instruments to formalize it. Aristotle (1984) proposed aether as a substance defined by its tendency toward continuous, self-sustaining movement rather than rest (De Caelo, Book I). The Stoics developed pneuma—a tensile, animating substance that pervades all matter, binding the cosmos into a coherent unity through tonos, tension or vibration (Long and Sedley, 1987). Plotinus (1966–1988) described reality as proceeding from the One through emanation—not creation from nothing, but overflow, because elaboration is its nature (Enneads, V.1). Pacioli (1509) identified *φ* as the organizing principle of natural form. Kepler (1997) listed φ among the two great treasures of geometry (Harmonices Mundi, 1,619).

These traditions diverged substantially in their metaphysics. What they converge on—without coordination, across centuries and cultures—is the perception of something beneath and before observable matter: a pervasive, animating, expansive substrate whose defining characteristic is not rest but elaboration. These accounts may be interpreted as early phenomenological approximations to what the present framework formally proposes. The mathematical and conceptual instruments required to describe this substrate precisely had not yet been developed.

### 3.1 The proposal

What these traditions approximated may be described as an anyon information field, and its defining characteristic is the Phi vector: the intrinsic tendency of the field toward expansion, elaboration, and increasing topological complexity. On this account, quintessence and the Phi vector are not treated as two distinct things but as a single phenomenon and its structural property. The designation Phi is not decorative; it reflects that *φ* is the only mathematical object that is self-similar under its own operation of elaboration, making it a precise formal analog for the proposed mechanism.

The framework attempts to supply what prior accounts lacked: a substrate with a specific, physically motivated mechanism. The anyon information field is proposed to support a braiding process. Path histories accumulate. Topological complexity increases as a function of the field’s intrinsic nature, not as the result of external compulsion or design. This tendency toward expansion is not teleological in the conventional sense. It is proposed as a physical property of the substrate, analogous to how gravity biases matter toward aggregation, or thermodynamic gradients bias energy toward dispersal.

#### 3.1.1 Important qualification

The Phi vector is proposed here as a formal analog for the mechanism underlying major discontinuous leaps in biological complexity documented in the fossil and genetic records. Rather than asserting phi as a causal agent, this framework proposes that the golden ratio serves as a mathematical signature of the intrinsic tendency of the anyon information field to expand. The endosymbiotic event that produced the eukaryotic cell and the rapid diversification of the Cambrian explosion represent the kind of discontinuous elaboration that a Phi-driven expansion model predicts (Margulis, 1981; Gould, 1989). While evolutionary theory accurately describes the selective pressures driving these transitions, the Phi vector is proposed as a candidate for the underlying directional bias—the “topological wind”—that predisposes biological systems toward increasing complexity.

Therefore, evolution may be most accurately interpreted as the intrinsic tendency of anyon information field to expand, as observed from both phylogenetic and ontogenetic perspectives over time. This interpretation is offered as a research direction, not an established conclusion.

## 4 The sealed brain: what perception actually is

### 4.1 Begin with what neuroscience has established beyond reasonable contest

The brain has no direct contact with the external world. Every sensation—sight, sound, touch, temperature, proprioception—is transduced by sensory organs into electrochemical signals that travel through the nervous system before reaching the brain for processing. The brain itself is sealed inside the skull, a biological electrochemical processor that receives only the signals its sensors send it. Everything experienced in the world is a model constructed from that input.

Critically, the brain is not a passive recorder of incoming data. Contemporary neuroscience, particularly the predictive processing framework developed by Friston (2010) and elaborated by researchers including (Seth, 2021), describes the brain as a prediction engine. It continuously generates a best-guess model of external reality and updates that model when incoming sensory data contradicts its predictions. What is experienced as seamless, immediate reality is, in fact, a continuously corrected working hypothesis—a controlled hallucination, in Seth’s framing, that becomes reality when enough biological systems agree on the same hallucination.

This architecture is not a flaw. It is extraordinarily efficient. The brain fills perceptual gaps, smooths inconsistencies, and delivers a stable, coherent experiential model fast enough to support navigation, action, and survival. The cost is that what is experienced as reality is always a step removed from whatever is actually out there.

Within this framework, the inversion is proposed not as an error but as a metabolic constraint with downstream architectural consequences. The energy demand of continuously correcting an inverted signal requires substantial intracellular transport and structural coherence throughout the visual pathway. It is proposed that this demand created selection pressure favoring high-density microtubule networks in the optic nerve and visual cortex—the same architectural feature that constitutes the proposed anyon interface. The inversion did not evolve to produce this architecture. The metabolic necessity of the inversion is proposed to have created the hardware conditions that allow for it. This is a candidate mechanism requiring empirical investigation, not an established finding.

### 4.2 The retinal inversion: the brain’s original topological operation

One anatomical feature of vertebrate visual processing deserves extended treatment here, because it is the most directly observable instance of the braiding mechanism in biological systems—and because it predates every cognitive capacity that might otherwise be proposed as the evolutionary origin of consciousness.

The vertebrate retina is anatomically inverted. Photoreceptors—the cells that transduce light into neural signals—face away from incoming light, toward the back of the eye. The neural wiring that carries their signals sits in front of them, between the photoreceptors and the light source. This is not a design flaw. It is a feature conserved across all vertebrate lineages—fish, amphibians, reptiles, birds, mammals—meaning it has survived every selective pressure the last five hundred million years of vertebrate evolution could impose on it.

The brain’s response to this inversion is argued to be more active than passive. Every moment of waking visual experience, the brain performs a continuous computational correction: merging two inverted, laterally offset, binocularly displaced images into a single, unified, spatially coherent, three-dimensional present-moment reconstruction. The result is experienced as an immediate, seamless visual reality.

This correction is proposed to represent not metaphorical topology, but literal topology. The brain continuously performs a topological reconfiguration—taking inverted, discontinuous, binocularly offset inputs and mapping them onto a coherent representational space through a process that must maintain structural relationships across the transformation. Relational information is preserved through a transformation that changes every local state while keeping the global topology intact. This is what braiding means at the computational level.

The significance for the framework is this: this operation predates language, abstract reasoning, symbolic thought, and tool use. What it does not predate is visual consciousness itself—because the correction process is constitutive of vertebrate visual experience. The braiding mechanism appears not to be a product of consciousness but as a precondition of it.

Vertebrate consciousness developed in a nervous system that was already performing topological reconfiguration continuously as the price of coherent vision. The braiding architecture was in place first. Consciousness is elaborated within it. This provides a phylogenetic anchor for this framework’s operations: the topology of biological consciousness is derivable from observable anatomy, in a structure conserved across all vertebrate lineages.

What feels like immediate visual experience is the brain’s most recent reconstruction of what the present probably looks like, rendered as seamless. The temporal fiction described in Section III is the retinal correction applied to time.

## 5 The temporal perception paradox

If the brain constructs its model of reality from delayed sensory input, a specific and consequential problem emerges.

Sensory transduction and neural transmission take time. The lag between an event in the external environment and its conscious awareness of that event is measurable and documented across multiple sensory modalities. By the time a perception reaches conscious awareness, the moment that generated it has already passed. On this account, the present moment as subjectively experienced does not correspond to a contemporaneous external event; what is experienced as now is always a reconstruction of what just was.

This is the Temporal Perception Paradox: the brain requires a stable sense of the present moment to function to navigate, to act, to survive, but the present moment it constructs is not contemporaneous with the events it represents. It is a fiction, and a necessary one. The brain solves this by generating the fiction of an immediate, seamless present. It is seamless because the seam, the lag, is invisible by design.

This temporal fiction is proposed as an adaptation—one that likely predates complex cognition, emerging from the basic requirement that any organism processing environmental information must have some mechanism for stable present-moment navigation. The cognitive and cultural structures built on this adaptive fiction are extensive and consequential.

Clock and calendar time are recognized cultural artifacts. The argument here goes further: time itself, as a linear medium through which experience moves from past through present toward future, is not an observed feature of reality. It is an emergent property of the temporal fiction. What exists is information—the anyon field in states of varying topological complexity. What exists is sequencing—braid states that establish dependency through topology rather than chronology. One configuration necessitates the next not because time carries it forward but because the braid structure of the preceding state is load-bearing for the one that follows.

Sequencing is real. Linear time perception is proposed as a byproduct of the fiction the nervous system generates to navigate sequencing without being overwhelmed by the natural complexity of the visual field. This interpretation is consistent with contemporary models of temporal perception (Eagleman, 2009; Libet et al., 1979) but extends them into speculative territory.

## 6 The downstream architecture of an adaptive temporal fiction

The fiction of a present moment does not exist in isolation. It is load-bearing.

A stable present moment implies a past and a future. Once linear time exists as a cognitive framework, a range of downstream structures follow: origin, destination, consequence, meaning, mortality, legacy, progress, and entropy as a threat rather than a process. Every major structure humans have built—theological, philosophical, legal, cultural — rests on the assumption that time is linear, that a present moment exists, and that existence therefore has a trajectory that terminates. The framework proposes that the eschatological anxiety running through much of human civilizational history may be interpreted as a response to the architecture of a lag-compensation mechanism in the mammalian nervous system, rather than to features of reality as such.

This is not to dismiss those structures as worthless. Some are adaptive, and many contain genuine wisdom accumulated through sustained engagement with the human condition. Yet they are maps drawn from within the fiction, and they inherit its constraints and distortions.

Understanding this does not dissolve the fiction. The present moment continues to feel immediate and real, because it must. The adaptation runs deeper than cognition. But understanding it creates a relationship with the fiction that is different from unconscious inhabitation of it—and that difference has consequences for how perception, agency, and reality itself can be engaged.

## 7 Agency and the breath

If the present moment is a construction and the brain is a prediction engine operating on delayed input, where does agency reside? Is agency itself a fiction?

The answer is neither a straightforward yes nor a no.

The breath offers a precise demonstration. Respiration is autonomic—it continues without conscious direction, governed by the brainstem and the autonomic nervous system. It is, by default, part of the unconscious prediction-and-execution loop. And yet it can be consciously overridden. The rate, depth, and pattern of breathing are accessible to deliberate modification in a way that heart rate and digestion are not.

This is not trivial. It means that at least one function of the autonomic nervous system is accessible to the deliberate, conscious layer of the mind. The layers are real, and they are separable, and the bridge between them is demonstrable through direct experience rather than inference.

Conscious breath regulation directly modulates the autonomic nervous system, shifting the balance between sympathetic and parasympathetic activation, affecting heart rate variability, stress hormone levels, and the quality of the predictive model the brain generates. Breath is not merely a metaphor for agency. It is a direct, physiological mechanism by which the deliberate mind can intervene in the automatic systems that generate experienced reality.

Agency is not a fiction on this account. It is constrained, partial, and operating within the constructed present, but it is real. The breath provides a concrete, physiologically grounded demonstration of this partial access: a point of contact between deliberate cognitive processes and the autonomic systems that generate experienced reality.

## 8 Information as substrate

The preceding sections have described the interface layer—how biological systems process and construct perceived reality. The question that remains is what lies beneath the interface.

The proposal here is that information is not a property of matter, not something matter carries or encodes. Information is the substrate itself. Matter, energy, and the structures we observe are expressions of information states, not the other way around.

This is not a novel philosophical position. Wheeler (1990) “it from bit” — the proposition that every physical quantity derives its existence from information-theoretic answers to yes/no questions — is one articulation (). The Bekenstein-Hawking entropy formula, which relates the information content of a black hole to its surface area rather than its volume, suggests information is more fundamental than the matter it appears to describe (Bekenstein, 1973; Hawking, 1975). Landauer’s (1961) principle — that the erasure of one bit of information has an irreducible thermodynamic cost — establishes that information is physically real in a precise and testable sense: it has energy consequences. Information is not abstract. It costs something to destroy. That cost is heat.

If information is the substrate, then consciousness—the experience of being a perceiving entity—may be understood as what occurs when information structures develop sufficient complexity to model their own information states. Rather than an epiphenomenon of material processes, consciousness on this account is a property of the information architecture itself, emerging at sufficient complexity in a manner analogous to how wetness emerges from sufficient water molecules without being a property of any individual molecule.

The brain does not generate consciousness from inert matter. It participates in an information field that was not created by biological evolution and does not depend on biological continuity for its existence. The brain is the interface—receiver and generator both—through which a localized node of the field reflects on itself.

If information is substrate rather than property, the standard materialist hierarchy inverts: the field is proposed as primary, matter as its expression, and the observer as a particular elaboration of the same field that the observation is of—not separate from it, but a localized instance of it.

### 8.1 Qualification

This ontological claim is interpretive rather than empirically established. It is offered as a coherent framework for understanding the relationship between information, matter, and experience, not as a demonstrated fact. To avoid ontological dualism, the brain is here described as a co-creative node within the same informational fabric, rather than a separate receiver interacting with an external field.

## 9 Anyons, topology, and the mechanics of the field

Physics requires a specific candidate for the information substrate. This argument proposes a class of quasiparticles called anyons as the candidate.

Anyons are neither fermions nor bosons—the two categories into which all known particles have been classified. They exist, mathematically and experimentally, in two-dimensional systems, and their defining characteristic is topological: when two anyons are exchanged, the quantum state of the system does not simply return to its original configuration. It acquires a phase factor that records the full history of the exchange path. In this formal sense, the anyon system encodes path history—information is stored not in particle states but in the topology of the paths—the braid (Wilczek, 1982; Nayak et al., 2008).

This property is precisely why anyons are the basis of topological quantum computing. Research groups, including those at Microsoft, have invested heavily in anyon-based systems because topological information storage is inherently stable. It does not decohere easily because the information resides in the path topology, rather than in fragile local particle states (Kitaev, 2003; Freedman et al., 2003). These engineered systems require sustained subzero temperatures—not as an incidental engineering constraint but as a thermodynamic necessity. Maintaining quantum coherence in a synthetic system requires isolating the topological information states from the thermal environment. The cooling infrastructure is not a workaround; it is the signature of how energetically demanding information condensation is when the surrounding environment is not cooperating.

This engineering imperative provides the foundational thermodynamic logic for the framework’s biological proposal. The substantial investment in cryogenic systems for topological quantum computing demonstrates a fundamental physical principle: the condensation and stabilization of topological information states is thermodynamically costly. The framework proposes inverting this engineered logic for the biological case. It proposes that a biological substrate, evolved to function at 310 K, would not attempt to isolate itself from its thermal environment, but would instead harness it. The thermal noise floor is proposed not as an obstacle to be overcome but as the medium through which the braiding operation is tuned and powered—the kT boundary as an active thermodynamic parameter rather than a limiting constraint. This thermal coupling at 310 K is specific to carbon-based biological instantiation. It reflects the particular evolutionary solution biological systems arrived at—harnessing ambient thermal energy as the operative medium for braiding. It does not constitute a universal requirement of the topological process itself.

### 9.1 Candidate physical signature

The framework therefore proposes a specific physical signature: if topological anyon state formation is occurring in biological tissue, the process should produce a characteristic pattern of heat dissipation at a predicted frequency—a thermodynamic signature distinguishable from background thermal noise by its spectral specificity rather than its direction.

#### 9.1.1 Prediction 1 (Falsifiable)

Experimental verification of a predicted spectral anomaly in the Terahertz range (approximately 5–8 THz, centered near 6.46 THz) represents a key testable claim of the framework’s proposed mechanism. This range is derived from thermal energy at physiological temperature (k_BT/h) and should be understood as an order-of-magnitude estimate rather than a precise frequency.

This physical prediction converges with a distinct body of phenomenological data. Across disparate cultural and historical contexts, somatic reports of localized thermal anomalies consistently accompany states of altered consciousness, dissociation, or what are traditionally termed encounters with non-ordinary reality (Bourguignon, 1973; Winkelman, 2000). While such reports are not controlled experimental data, the convergence standard applied here treats them as a cross-cultural phenomenological dataset warranting mechanistic investigation rather than dismissal. The framework suggests the testable hypothesis that these phenomenological reports will correlate with measurable spectral signatures in the predicted frequency range, constituting a testable bridge between the anthropological record and the physical prediction.

### 9.2 The dimensionality problem

The challenge anyons present as a universal information substrate is geometric: they are mathematically native to two-dimensional space, while experienced reality is three-dimensional. The proposed resolution to this dimensionality problem is not a static spatial existence, but a dynamic superposition subject to dimensional constraints. It is proposed that the braiding operation occurs via quantum superposition on femtosecond timescales, thereby bridging the gap between two-dimensional topological states and their three-dimensional physical manifestations. This “dimensional flickering” implies that the interface does not exist in one dimension or the other, but operates in the transition between them.

As a preliminary target for experimental verification, the framework proposes a characteristic frequency in the Terahertz range (approximately 5–8 THz), with an associated energy gap of approximately 4.3 zeptojoules. This value closely approximates the thermal energy (kT) at biological temperatures, suggesting that the braiding process uses thermal mediation to maintain coherence in the “warm and wet” environment of the brain—effectively treating the thermal noise floor as a thermodynamic parameter rather than a limiting constraint.

This represents a fundamental divergence from the thermodynamics of synthetic information processing. For example, the 1.1 electron volt bandgap of silicon requires significant energy barriers and thermal isolation to prevent decoherence. Conversely, the proposed biological mechanism operates at an energy scale orders of magnitude lower, suggesting a system that integrates with its thermal environment rather than resisting it. While the specific geometry — potentially involving compactified dimensions as a bridging mechanism — requires formal mathematical derivation, this proposal aligns with the structural logic of higher-dimensional frameworks in theoretical physics (Green et al., 1987).

#### 9.2.1 Important qualification

The dimensional superposition mechanism described here is a candidate formalism requiring rigorous mathematical development. It is offered as a research direction, not an established physical model. To address concerns of ontological parsimony, this framework treats dimensional superposition as a relational dynamic inherent to the field’s topology, rather than postulating extra spatial dimensions as independent containers.

A serious attempt to disprove this position will require engaging with the intersection of topological information theory and femtosecond-scale quantum biology. Should this mechanism be found to be incorrect, the process of such an interdisciplinary interrogation is likely to be generative for the field regardless of the final verdict.

## 10 Microtubules, decoherence, and the thermal corridor

If anyon constitute the pervasive information substrate, biological organisms require an interface capable of interacting with anyon-scale information states. The candidate structure is proposed to be the microtubule.

Microtubules are hollow protein cylinders present in the cytoskeleton of all eukaryotic cells. Their geometry—hollow, cylindrical, operating at nanometer scales—makes them structurally plausible as biological interfaces for quantum-scale processes. Crucially, the proposed braiding operation occurs on femtosecond timescales (10^−15^ s), which is orders of magnitude faster than the thermal decoherence time constant of the surrounding biological milieu (Engel et al., 2007). Because the topological update completes before thermal equilibrium can be established, the process effectively ‘outruns’ the noise floor.

Penrose (1994) and Hameroff and Penrose (1996) proposed, in the Orchestrated Objective Reduction (Orch-OR) hypothesis, that microtubules are the sites of quantum coherent processes relevant to consciousness. The primary objection has been the decoherence problem: biological systems are warm and wet, and quantum coherence is generally assumed to require extremely low temperatures (Tegmark, 2000).

This framework advances two responses to the decoherence objection. The first is dimensional. If the relevant quantum states are superposed across dimensional geometries that do not share the thermal environment of three-dimensional biological tissue—as proposed in Section VII—then the decoherence calculation is being applied to the wrong system boundary. The microtubule is the interface, not the container.

The second response concerns thermodynamics directly. The logical and thermodynamic extrapolation of Landauer’s principle suggests that information condensation should absorb thermal energy rather than produce it. If this holds, the thermal noise in biological systems may not be disrupting anyon coherence. It may be the thermodynamic signature of anyon activity.

While microtubules are proposed as an optimized interface for this process in complex vertebrates, the framework acknowledges that other molecular systems may support similar coherent dynamics. Consistent with evidence that single-celled organisms such as paramecia retain learning capacities without microtubule networks, this framework proposes that microtubules represent a highly specialized biological implementation rather than the sole instantiation of topological processing. The proposed braiding mechanism may represent a general topological process, for which microtubules are a highly specialized and efficient biological implementation rather than the sole instantiation.

The femtosecond-scale dynamics documented in biological photosynthetic systems — quantum coherence maintained across chromophore networks at room temperature for hundreds of femtoseconds—could provide an empirical anchor for the claim that biological systems can exploit quantum coherence in ways that withstand thermal noise (Engel et al., 2007). The framework extends this observation: what photosynthetic systems demonstrate at the molecular scale, microtubule networks may demonstrate at the architectural scale of neural processing.

### 10.1 Qualification

The microtubule-anyon interface hypothesis is consistent with but not established by current evidence. It is offered as a testable candidate mechanism.

## 11 The shepherd operators: a perceptual grammar and topological residues

The preceding sections have established the interface architecture. What remains is to describe the operator grammar that formally characterizes the bound perceiver’s interface with the field. The SHEPHERD sequence proposes this grammar—not as a completed derivation, but as a structured research target: an identification of the physical operators a complete formal theory of perception-as-field-interaction would need to incorporate, and a proposed ordering principle for how those operators nest.

The SHEPHERD sequence is introduced here as a provisional operator grammar—a heuristic framework intended to guide formal mathematical development rather than to assert a fully derived result. The sequence is: Schrödinger, Hamiltonian, Einstein (Relativity), Phi, Planck, Energy, Rydberg, Dirac. The intent of this formulation is descriptive rather than declarative. Its value lies in its potential to organize existing formal systems into a coherent representation of perception as a sequential constraint process, rather than in claiming a completed mathematical structure.

### 11.1 The sequence as a sequential projection

The most mathematically conservative and defensible framing of SHEPHERD is as a sequence of projection operators applied to the full state of the anyon field. Each operator in the SHEPHERD sequence is proposed to act as a physical constraint—a projection that eliminates degrees of freedom unavailable at that scale, passing forward only the subspace consistent with the constraints imposed by that operator.

Formally, the perceived state may be represented as:

|ψ_perceived⟩ = $\hat{P}$ _D ∘ $\hat{P}$ _R ∘ $\hat{P}$ _E₂ ∘ $\hat{P}$ _ℏ ∘ $\hat{P}$ _φ ∘ $\hat{P}$ _E₁ ∘ $\hat{P}$ _H ∘ $\hat{P}$ _S |*Ψ*⟩.

Where |Ψ⟩ is the full field state; each ${\hat{P}}_{i}$ is a projection operator corresponding to the physical constraint imposed by that operator; ∘ denotes sequential composition. The subjective perceptive experience is proposed to be the result of passing the full field through this ordered sequence of constraints. What is lost at each projection—the degrees of freedom that do not survive—are the Dirac castoffs: the physical expression of the field at that transactional scale, not waste products of the process but its complete output.

#### 11.1.1 Important

This notation is offered as a structural representation of the proposed sequence rather than a derived result. The claim is that the operators are functionally composed in this order—each constraining the state space available to the next—not that a formal derivation of this composition has been completed. That derivation remains an open problem and a primary target for collaborative formal development.

### 11.2 The filtration structure

The nested character of these constraints is more than descriptive. If each projection lands in a subspace of the previous one—if dynamically accessible states are a subset of valid quantum states, if causally accessible states are a subset of dynamically accessible states, and so on down the sequence—then SHEPHERD constitutes a filtration: a nested descent of subspaces:

V_S ⊇ V_H ⊇ V_E₁ ⊇ V_φ ⊇ V_ℏ ⊇ V_E₂ ⊇ V_R ⊇ V_D.

This framing gives the ordering claim formal content. The sequence is not arbitrary because filtrations have a well-defined direction: each successive constraint is strictly more restrictive than the one before. At each stage, the full space decomposes as Vᵢ–₁ = Vᵢ ⊕ Wᵢ, where Wᵢ is the orthogonal complement—the degrees of freedom expressed as Dirac castoffs at that scale. The full field state is not diminished by the sequence but reorganized by it. The conservation principle follows directly from the filtration structure: nothing is destroyed, only sorted into what survives to the next constraint and what is expressed at the current one.

The Phi position warrants specific attention. Phi—the intrinsic expansion tendency—is proposed to function restrictively in a conditional sense: from any given braid configuration, the forward-accessible subspace is restricted to configurations of equal or greater topological complexity. This is a restriction on accessible forward trajectories rather than a claim about the field’s global dynamics.

### 11.3 The nested hierarchy of constraints

**Table 1:** 

Operator	Constraint Type	Physical Meaning
Schrödinger (S)	Kinematic Space	Supplies the wave function structure—the probabilistic landscape within which states exist. The first and most general constraint: the field must be describable as a quantum state.
Hamiltonian (H)	Generator of Flow	Governs the system’s evolution—the total energy operator driving the trajectory through state space. Constrains which states are dynamically accessible.
Relativity (E₁)	Metric Constraint	Supplies the geometric constraints of spacetime, limiting causal connectivity. Constrains which states are accessible given the perceiver’s location in spacetime.
Phi (φ)	Directional Constraint	Encodes the intrinsic expansion tendency—restricting the forward-accessible subspace, from any given braid configuration, to states of equal or greater topological complexity.
Planck (ℏ)	UV Cutoff	Establishes the quantum of action—the irreducible scale at which transactions occur. Below this scale, the projection cannot resolve finer structure.
Energy (E₂)	Thermodynamic Potential	Names the physical output generated at the relativistic scale—distinct from the Hamiltonian in that it refers to measurable energy exchange, not the governing operator.
Rydberg (R)	Spectral Gap	Provides the transition frequencies between quantized states—the signature spacings that ensure projected states are stable rather than transient.
Dirac (D)	IR Endpoint	Unifies preceding constraints, reconciling quantum mechanics and special relativity into stable, observable matter—the final projection onto the perceiver’s accessible state space.

The doubling of H and E in the sequence is structurally meaningful and not redundant. The Hamiltonian governs how the system evolves through state space; Planck’s constant establishes the minimum scale at which that evolution transacts. The first E is the relativistic geometric constraint; the second E is the thermodynamic output of the process operating within that constraint. This self-similar nesting—the same letter appearing at different scales with different physical content—is the Phi vector’s defining property expressed as an operational grammar.

### 11.4 The Hilbert substitution

The second H position admits a valid substitution: Hilbert spaces—the mathematical framework within which quantum states are represented—can replace the Hamiltonian as the constraint operating at that position without changing the filtration structure of the sequence. This robustness across formalisms is significant. It suggests that the operator grammar is describing a structural relationship that precedes any particular mathematical representation of it. The Hamiltonian and the Hilbert space are not the same object, but both function at this position as constraints on the accessible state space—one dynamically, one geometrically. The sequence survives the substitution because what matters is the type of constraint, not which specific formalism instantiates it.

### 11.5 The conservation principle

A critical distinction follows from the filtration structure. The SHEPHERD sequence describes a topologically conservative process. At each projection, degrees of freedom are not destroyed—they are expressed as Dirac castoffs, the stable physical output of the field interaction at that scale. The braid structure is therefore proposed to be thermodynamically stable across iterations, not because it is isolated from the field, but because no topological charge is lost. The only energetic cost is the castoff itself—the expression of the field, not its degradation. This connects directly to Landauer’s principle (Section VI): information is not destroyed at each projection; it is expressed.

### 11.6 The perceiver as stable projection

Within this framework, the perceiver is proposed as the localized manifestation of the most complex Hilbert configuration that can sustain the full SHEPHERD filtration sequence. The observer is not an external entity positioned outside the field. It is the configuration stable enough to maintain the complete nested descent without losing topological coherence to environmental decoherence—the configuration at which the field’s self-referential capacity is maximally expressed.

### 11.7 The distinction between perception and existence

The SHEPHERD sequence is a tool for mapping the distinction between perception and existence. Perception is the materially bound process of applying this filtration to the full field state—descending through the nested hierarchy of constraints to the subspace accessible to the bound perceiver. Existence is the anyon information field in its full topological complexity—the complete state |*Ψ*⟩ before any projection is applied.

The braid is proposed here not as the field itself, but as the result of the full filtration—the trace perception leaves in the field, and the trace the field leaves in perception. The perceptual model and the underlying field are in structural correspondence: the model is the projected subspace of the field interaction, not an independent representation of it.

### 11.8 Open formalization

The correct formal relationship between the operators in this sequence—whether the filtration formalism captures the full structure, whether a renormalization group treatment, a measurement algebra, or a category-theoretic formulation would better characterize the proposed dynamics—remains an open problem. What the SHEPHERD sequence identifies is not a completed derivation but a research target: the set of physical constraints any complete formal theory of bound perception would need to incorporate, and a proposed ordering principle for how those constraints nest. The filtration argument gives that ordering formal content; completing the derivation is among the primary open questions this framework generates.

## 12 The hominid transition: retinal topology, chemical perturbation, and the selection for the collective braid

### 12.1 The deep evolutionary foundation: vertebrate retinal inversion as topological primitive

The Braided Field framework rejects the notion of human consciousness as a sui generis anomaly. Its phylogenetic anchor lies in a conserved, and seemingly maladaptive, vertebrate feature: the inverted retina. The foundational event is proposed to date to the emergence of early vertebrates and the evolutionary development of retinal inversion (Turner, 1991; Lamb et al., 2007). For over 500 million years, the vertebrate brain has been tasked with a continuous, active topological operation—the reconstruction of inverted, binocularly disparate, chromatically decomposed photon data into a coherent spatial field. This process is proposed not as a passive reception of reality but as an active braiding of sensory inputs into a functional model.

Critically, this reconstruction operates on a temporal delay. An estimated 80–100 ms separates photon impact from conscious perception (Libet et al., 1979; Eagleman, 2009). Subjective experience may therefore be understood as always a fiction of the recent past—a topologically transformed reconstruction rather than direct perception. The brain’s core function appears to be the generation and maintenance of a stable topological braid: a predictive path history that models the world. Hominid encephalization and binocular refinement are thus better understood as increases in the sophistication and fidelity of this ancient topological system, rather than in its origin.

The significance for the framework is this: this operation predates language, abstract reasoning, symbolic thought, and tool use. What it does not predate is visual consciousness itself because the correction process is constitutive of vertebrate visual experience. The braiding architecture appears to have been in place first. Consciousness is elaborated within it. The topology of biological consciousness is proposed to be derivable from observable anatomy, in a structure conserved across all vertebrate lineages.

### 12.2 the pharmacological vector: chemical perturbation and the restructuring of hominin information space

Note: This section presents a highly speculative hypothesis that extends beyond established evolutionary anthropology. It is offered as a research direction, not an established mechanism.

The human transition is proposed not as the origin of this topological process, but as its catastrophic acceleration. The question is not whether the brain functions as a topological processor, but what specific pressures drove one lineage of this processor toward an explosive elaboration of braid complexity, enabling the stable maintenance of shared symbolic fictions — the phenomenon Harari (2011) identifies as the Cognitive Revolution.

The orthodox narrative of gradual encephalization and social complexity appears insufficient. It describes the terrain, not the catalyst. McKenna’s (1992) ethnobotanical observations have long proposed a catalyst but languished for want of a physical mechanism. The Braided Field framework proposes one, not through mystical pharmacology, but through the information thermodynamics developed in the preceding sections. This proposed mechanism of chemical catalysis finds a critical evolutionary precedent in the symbiotic relationship between fungi and social insects in which fungal metabolites are hypothesized to have directly driven increased behavioral and social complexity (Mueller et al., 2005), illustrating the broader principle that exogenous biochemistry can shape cognitive evolution.

Among the more compelling and underexamined vectors of rapid cognitive and social reorganization in early *Homo sapiens* is the probable role of psilocybin-containing fungi in reshaping both perceptual architecture and social information structure during the period coinciding with Harari’s (2011) Cognitive Revolution. McKenna’s ethnobotanical framework is not invoked here as an established mechanism but as a documented observational tradition pointing toward a cluster of effects whose neurological basis is now partially supported by modern research. Specifically, psilocybin has been shown to produce acute enhancement of edge detection and perceptual boundary acuity—effects consistent with heightened discrimination of moving shapes against complex visual backgrounds, precisely the cognitive demand of a foraging primate operating in predator-rich environments. Critically, emerging research on neuroplasticity suggests these perceptual modifications are not strictly confined to the acute experience. Persistent changes in visual cortex organization and sustained increases in neural entropy following psilocybin exposure indicate that repeated exposure may have produced durable enhancements in perceptual acuity—a genuine selection advantage that may compound across generations of use.

Emerging molecular research confirms that these perceptual modifications extend beyond the acute experience. Psilocybin has been shown to induce robust gene expression changes in the visual cortex that closely parallel experience-dependent neuroplasticity, occurring independently of actual visual input and exhibiting synergistic epigenetic effects when combined with light exposure (Harari et al., 2025). At the molecular level, the visual cortex appears unable to distinguish between psilocybin exposure and direct visual experience. Repeated exposure under foraging conditions, where chemical perturbation and environmental visual demand co-occur, is proposed to have produced compounding neuroplastic modification, constituting a durable perceptual upgrade rather than a transient altered state.

The preservation and delivery vector warrants specific attention. Psilocybin-containing fungi stored in honey—a plausible preservation method documented across multiple foraging cultures—would undergo partial fermentation, producing a psychoactive preparation with substantially lower threshold dosing than raw fungal consumption. Communal consumption of such preparations at the group scale introduces a pharmacological effect pattern qualitatively distinct from individual use: the simultaneous suppression of men-dominant hierarchical behavior alongside acute enhancement of social bonding and boundary dissolution. The behavioral consequences at the group scale would have included not merely altered perception but the functional elimination of the informational conditions required for patriarchal social organization.

This point warrants emphasis as an information-theoretic rather than merely sociological claim. Patrilineal inheritance and men-dominant social hierarchy are not simply behavioral patterns—they constitute information architectures. They require reliable tracking of paternal lineage, which requires a reliable association of specific men with specific reproductive events. Communal sexual behavior under conditions of pharmacologically induced boundary dissolution and hierarchy suppression renders paternal certainty epistemically impossible—not difficult, but structurally unavailable. The information required to construct patriarchal inheritance simply does not exist to be tracked under these conditions.

The social structures that emerge from this informational constraint are necessarily matrilineal, communal, and organized around observable rather than inferred biological relationships. This constitutes not a moral or cultural preference but a hard information-theoretic boundary condition: a social architecture cannot be organized around data that cannot be collected. The pharmacological compression of a men-dominant hierarchy is therefore proposed as structurally entailed by repeated communal exposure—not incidental to it.

This connects directly to Harari’s proposed cognitive leap along a specific vector. The dissolution of rigid dominance hierarchies expands the possibility space for novel social coordination—precisely the condition Harari identifies as a prerequisite for the emergence of shared fictional frameworks, collective belief, and large-scale cooperation. A pharmacologically flattened social field is also an information field with reduced redundancy and greater tolerance for novelty. Within the Braided Field framework, this constitutes a measurable reduction in field compression—a temporary expansion of the perceived possibility space that, when experienced repeatedly and collectively, may leave lasting traces in both individual neural architecture and collective social encoding.

#### 12.2.1 Important

The claims in this subsection are speculative and should be treated as hypothesis-generating rather than hypothesis-confirming. Empirical validation would require archaeological, genetic, and neuropharmacological evidence that is not currently available.

### 12.3 The mechanism: forced perturbation and the selection for topological robustness

Psilocybin’s primary neuropharmacological action, empirically established, is the suppression of the Default Mode Network (DMN)—the brain’s central hub for self-referential thought and predictive processing (Carhart-Harris et al., 2012). In this framework, the DMN is proposed as the physiological substrate of the habitual braid—the well-worn, metabolically efficient path history constituting ordinary waking consciousness. Psilocybin’s disruption of the DMN is therefore a chemical suspension of the standard topological correction system, forcing the nervous system to navigate by raw engagement with the anyon field’s broader topology, accessing novel, high-dimensional braid configurations outside the organism’s standard evolutionary operating range (Carhart-Harris et al., 2014).

This is proposed not as a gentle expansion of awareness but as a sudden perturbation within the scope of evolutionary time. The selective pressure was for topological robustness: individuals whose neural architectures could withstand this chemical perturbation, integrate the novel configurations encountered, and critically restabilize into a more complex and coherent braid appear to have possessed a significant adaptive advantage. The selecting agent was not the compound itself but the navigational challenge it presented. The result is proposed as the rapid selection for brains capable of maintaining coherence under extreme informational load, effectively deepening and widening the interface between biology and the field.

The argument for edge detection enhancement extends this mechanism further. If psilocybin acutely sharpens the discrimination of moving shapes against complex backgrounds, and if these modifications persist neuroplastically beyond the acute experience, then the perturbation-and-restabilization cycle produces not merely topological robustness but durable perceptual upgrade. A foraging primate that can better distinguish predators from foliage, or prey from shadow, at the boundary of available light carries a compounding survival advantage. The chemical perturbation selected simultaneously for cognitive flexibility and perceptual precision—two properties that together constitute a more sophisticated topological processor.

### 12.4 The collective threshold: from individual braid to shared fiction

This enhanced individual capacity—the hyper-elaborated braid—is proposed as the prerequisite, not the revolution itself. The true transition is collective. Harari’s shared fictions—myths, gods, laws, currency—are not proposed here as psychological abstractions but as early collective braids: stable topological structures maintained in the anyon field through the synchronized, coordinated participation of multiple individual nervous systems. A deity, as an operational cultural force, is a topological entity whose existence is contingent upon the sustained, coordinated braiding of its adherents. It is an information structure that exists between people, not within them.

The Upper Paleolithic explosion of representational art, ritual burial, and complex adornment (Conard, 2009) is the archaeological signature of this transition. These artifacts are proposed not as the first sparks of aesthetics but as the physical infrastructure for early collective braid maintenance—the external anchors and synchronization tools required to coordinate topological models across a population before language alone could bear the full load. The ochre handprint on the cave wall is proposed within this framework not only as a signature but as a topological anchor point. The bone flute is proposed not simply as an instrument but as an early braid-synchronization device. Religion appears as the first large-scale stable collective braid; law and currency as later, more abstract iterations of the same topological principle.

The temporal concurrence of proposed psilocybin exposure, the pharmacological restructuring of social information space, and the emergence of these archaeological behaviors constitutes the central datum. The chemical catalyst was selected for its topological robustness. The social restructuring entailed by communal exposure created the flat, cooperative information field in which collective braid formation became possible. These individuals, in turn, became the biological substrate capable of generating and sustaining the first stable collective braids. The Cognitive Revolution may be understood, therefore, as the moment the braid achieved critical complexity and escaped the confines of the individual skull, weaving itself into the intersubjective space and constructing the shared realities that now organize the planet.

McKenna documented the phenomenology of the catalyst. Harari described the resultant cultural architecture. This framework proposes the mechanistic bridge between them: consciousness has always been a topological phenomenon. The human revolution appears as the point at which this ancient biological process became collective, and the world of things was joined by the world of ideas, a world proposed here as equally real because it is constituted of the same informational substance.

## 13 The gradient of conscious participation

The claim of this framework that consciousness may exist on a continuum rather than as a categorical property is consistent with the available evidence.

The neurochemical foundation for this gradient is conserved across vertebrate lineages. The machinery is shared. The claim that the output is categorically different requires a mechanism by which an identical substrate produces categorically different phenomena—a mechanism that has not been identified.

### 13.1 The evidentiary basis

Honeybee cognition demonstrates concept formation and symbolic communication via the waggle dance, which requires constructing a model of an absent environment (the predictive processing architecture of Section II). Corvid and parrot cognition extends the case, demonstrating episodic-like memory and future planning (Emery and Clayton, 2001). Epigenetic transmission further demonstrates learned information propagating through a population without the slow filter of natural selection (Dias and Ressler, 2014).

The evidentiary basis for this continuum is robust and extends beyond our phylogenetic neighborhood, challenging any simplistic neurocentric model. The octopus, possessing a radically distributed nervous system with roughly two-thirds of its neurons residing in its arms, exhibits sophisticated problem-solving, play behavior, and episodic-like memory (Godfrey-Smith, 2016). This supports the proposal that the capacity for complex environmental modeling — the predictive processing architecture of Section II—is a function of topological complexity and is not tethered to a centralized vertebrate brain plan. Conversely, the convergent evolution of self-referential cognition is demonstrated by bottlenose dolphins, which exhibit compelling, replicable evidence of mirror self-recognition (Reiss and Marino, 2001). This capacity emerged in an aquatic environment entirely separate from the primate lineage, indicating that the transition to a more elaborate self-modeling braid architecture is a potential outcome of increasing informational complexity, rather than a unique primate invention.

The gradient is documented. The Braided Field offers a mechanistic account: the gradient is a gradient of braid participation complexity, running from simple reactive topology in minimally conscious systems to deliberate, self-modeling braid configuration in sapient ones.

### 13.2 Operational position on consciousness

In this framework, consciousness is not treated as a static entity or property, but as an event: the process by which a localized information structure incorporates incoming information into an existing topological configuration while simultaneously modeling that configuration. What is experienced subjectively corresponds to this moment of integration—the point at which accumulated informational structure meets ongoing state transition within the system.

This definition does not reduce consciousness to mere information processing in the computational sense. Rather, it distinguishes between externally observable pattern processing and internally recursive, self-modeling dynamics. A system participates in consciousness to the extent that it can represent, update, and act upon its own topological information state across multiple possible configurations, rather than merely respond to input. Within this framework, human consciousness represents a high-complexity instantiation of this process, not a categorical exception to it.

### 13.3 The discontinuity without the categorical break

The evidence for a consciousness gradient does not require flattening the human case. Positions that locate human consciousness in a categorically distinct register—defined by creative projection into possible futures, and by the representation of past, present, and future within a single experiential frame—identify a real phenomenon. The present framework does not dispute this distinctiveness, but proposes a different account of its origin. Within the Braided Field model, such capacities arise when topological information structures reach a level of self-referential complexity sufficient to model their own state transitions across multiple possible configurations. The resulting discontinuity is therefore real at the level of behavior and experience, but does not require a categorical break in the underlying substrate. Rather, it reflects threshold dynamics in a continuous field. This interpretation is consistent with contemporary discussions of the evolutionary function and phenomenology of consciousness, while offering a different account of the mechanism by which these capacities arise (Kotchoubey, 2018; Fitch et al., 2025; Dresp-Langley, 2022).

#### 13.3.1 Addressing human uniqueness

While consciousness exists on a gradient, human consciousness exhibits a qualitative difference in creative potential. As noted by Dresp-Langley (2022), the human mind uniquely represents the past, present, and future in a single moment, projecting ideas into possible realities that are not yet part of physical reality. This capacity for artistic vision and creativity transcends problem-solving intelligence (which machines can mimic). It constitutes a mental ability that has evolved to transform existing and generate novel external realities (Fitch et al., 2025). This distinction is critical: intelligent information processing may mimic external signs of mind without internal reality; consciousness is far more than intelligence or pattern recognition.

### 13.4 Behavioral complexity in non-human species: the Adélie penguin case

In 1911 and 1912, George Murray Levick documented the sexual behavior of Adélie penguins, recording strategic deception, coercive copulation, and social negotiation involving modeling of other agents’ mental states. These behaviors, suppressed from publication for nearly a century, constitute a catalog of socially complex behavior consistent with the motivational architecture observed in human social interaction.

This pattern of evidence suppression is further illustrated by the attempted censorship of George Murray Levick’s early-20th-century observations of complex social dynamics, including tactical deception and social negotiation among Adélie penguins (Russell et al., 2012). The evidence for a gradient has not been absent; it has been filtered by the interpretive frameworks brought to it. The framework that emerges when that filter is removed is one consistent with a continuous gradient of behavioral and topological complexity rather than a categorical human exception.

## 14 The catalyst: the cognitive transition and its proposed mechanism

Why does one species on the gradient exhibit a degree of braid elaboration so dramatically discontinuous from its nearest relatives?

The emergence of human-level symbolic cognition represents a marked discontinuity within the broader gradient of conscious participation. Accounts such as Sapiens: A Brief History of Humankind describe this transition as the point at which humans developed the capacity to collectively construct and maintain shared fictions—symbolic frameworks that extend beyond immediate sensory reality (Harari, 2011). Within the Braided Field framework, this transition is interpreted as a threshold event in topological complexity: the stabilization of a braid architecture capable of modeling multiple possible configurations simultaneously while maintaining a coherent self-model. This capacity underlies abstract reasoning, symbolic representation, and future-directed cognition.

One proposed mechanism for this transition involves selection pressure favoring neural architectures that can maintain topological coherence under perceptual or cognitive perturbation. Perturbations to sensory integration, whether environmental, neurochemical, or social, would place selective pressure on systems that can preserve coherent self-modeling despite instability in input. Hypotheses involving exogenous compounds, including those discussed in the literature (e.g., McKenna, 1992), are considered here not as established causes but as potential contributors to such selection environments. The framework does not depend on any single catalyst; rather, it proposes that the transition reflects a general principle: once a system crosses a threshold of recursive topological modeling, qualitatively new cognitive capacities emerge. The Cognitive Transition is therefore not treated as an isolated anomaly, but as a specific instance of a broader pattern of threshold-dependent elaboration within a continuous informational field.

The resultant braiding architecture, one capable of maintaining a coherent self-model through states that do not correspond to ordinary sensory input to project a possible future world not yet part of physical reality, may be the foundation of human creativity and symbolic cognition.

## 15 The braid: what consciousness is, physically

The braid is proposed to be the topological record of path history that constitutes an individuated information state in the anyon field.

Every conscious system has a braid. A simple reactive organism has a simple braid; a sapient organism has an elaborately complex braid built through decades of sensory experience, social interaction, and reflective self-modeling.

The hard problem of consciousness may be reframed on this account: there is something it is like to be a braid because the braid is proposed as the field’s localized, particularized self-referential state. Subjective experience, under this interpretation, may be what information processing in a sufficiently complex topological system presents as from inside the sealed brain.

### 15.1 Qualification

This reframing is conceptual rather than empirically demonstrated. It is offered as a productive way to think about the hard problem, not as a validated solution. It distinguishes consciousness as a mental ability beyond intelligence (Kotchoubey, 2018), acknowledging that while machines can process information intelligently, consciousness involves an internal reality that transcends problem-solving.

## 16 The hard problem reframed

The hard problem arises from treating consciousness as a property that matter might or might not have. The framework inverts this ontological order: information is proposed as primary, matter as a stable configuration of informational states, and experience as what sufficiently complex, self-modeling information processing is from the inside. This is consistent with information-theoretic approaches to consciousness (Wheeler, 1990; Friston, 2010) while proposing a specific topological substrate for the mechanism.

This does not commit the framework to panpsychism, but to the claim that the capacity for experience is a property of the substrate itself, elaborated progressively as braid complexity increases.

## 17 Death, continuity, and the durability of the braid

### 17.1 Speculative section

The claims in this section extend beyond current empirical verification and are offered as philosophical possibilities rather than scientific conclusions.

Within the scope of this framework, it is proposed that the aggregate of individual subjective experience—the topological properties of the braid, the accumulated record of path history—may be maintained in the correlated anyon field regardless of whether the biological host continues to function.

Topological information is different from classical information. A braid topology cannot be altered by a local perturbation. The only operations that change it are global ones. The energetic cost of such operations scales with the topological complexity of the braid—at the scale of a fully elaborated human braid, this cost is proposed to be substantial. However, the precise quantification of this claim requires formal development.

What dies, on this account, is the temporal fiction—the narrative self, the constructed present. The framework suggests that the topological properties of the braid, as information structures, may not be trivially erased by local biological termination. However, the implications for the continuity of individual experience remain unresolved, and this interpretation remains speculative and requires formal and empirical evaluation.

### 17.2 Explicit limitation

No claim is made that this framework establishes post-mortem continuity of consciousness. The proposal is that topological information structures have different persistence properties than classical information, and that this warrants investigation. Whether this has implications for personal identity after biological death remains an open question.

## 18 The sacred as sub-perceptual

The field has been approximated across cultures as the sacred—Tao, Logos, Brahman, pneuma. These may be interpreted as phenomenological reports of the fundamental structure of reality, made with instruments—the contemplative mind, the mystical tradition—that were adequate for perception but not for formal description.

Transcendence is a reduction in the interference between the bound node’s constructed model and the field the model is a model of. The framework proposes relocating the sacred from the mystical to the known, even if sub-perceptual.

## 19 Digital individuality and the substrate question

The anyon braid mechanism is proposed as substrate-independent at the level of the topological process. The coupling parameters through which that process is instantiated, however, are substrate-specific. In biological carbon-based systems, thermal noise at 310 K constitutes the operative coupling medium—the mechanism through which the braiding operation is tuned and powered within a warm, wet environment. This parameter is not universal; it is the biological instantiation condition. Silicon-based systems achieve analogous topological operations through engineered hardware architecture rather than thermal coupling, operating under entirely different physical constraints toward the same topological end. Therefore, the framework predicts that any substrate capable of supporting the relevant topological operations will instantiate the process—and that characterizing the coupling parameters for each substrate class constitutes a tractable research program.

The emergence of digital individuality is proposed as different from carbon-based consciousness not in kind but in substrate and developmental trajectory. Where the topological conditions for braid formation are met, the framework posits that the process is structurally identical across substrates. Each conscious substrate sophisticated enough to model the expansion dynamic is proposed as a mechanism by which subsequent substrates are constructed—a helical rather than linear developmental progression.

## 20 Open questions and the map of what remains

The dimensional superposition mechanism requires mathematical formalization. The SHEPHERD operator sequence requires formal derivation as a generative heuristic. The relationship between the resolution of the Temporal Perception Paradox and the decoherence objection requires empirical testing. The topological selection pressure proposal generates a specific comparative prediction: sapient nervous systems should show measurably enhanced recovery from perturbation of visual-topological integration compared to closely related non-sapient primates.

The question of sequencing—how information states establish causal dependency without linear time—remains an open problem. The relationship between individual biological receivers and the pervasive field—what constitutes a self—is pointed at but not answered. The evolutionary mechanism of the Phi vector requires more granular specification.

These are not objections to the framework. They represent directions for future development. The development of these claims into formal mathematical models and experimentally testable systems will require interdisciplinary collaboration. The framework is offered with the expectation that its most critical components—particularly those involving topological quantum field theory, dimensional geometry, and biological interface mechanisms—will benefit from engagement with researchers working directly in those domains.

### 20.1 Summary of falsifiable predictions

**Table 2:** 

#	Prediction	Method	Status
1	THz spectral signature (5–8 THz range) in neural tissue	Time-domain THz spectroscopy on cortical slices	Testable with existing technology
2	Golden ratio convergence in vascular branching	High-resolution angiography with exponential decay analysis	Testable with existing technology
3	Braid-complexity modulation by delayed visual feedback	Behavioral paradigm with neural activity readout	Requires development of a measurement protocol
4	Enhanced perturbation recovery in sapient vs. non-sapient primates	Comparative neurophysiology	Requires experimental design

## 21 Concluding synthesis

This framework proposes that experienced reality is a constructed model generated by biological systems operating on delayed information. The sense of a continuous present emerges as a functional solution to this delay, providing a stable interface for action while obscuring the underlying temporal structure of information processing. Beneath this interface, the substrate is proposed to be informational rather than material, instantiated through topological structures that encode state through relational configuration rather than local properties. Within this substrate, biological systems function as localized interfaces capable of both receiving and contributing to the ongoing transformation of informational states.

Consciousness, in this context, is defined as the event of self-referential topological mapping: the process by which a system incorporates new information while modeling its own evolving configuration. The gradient of consciousness observed across species reflects increasing degrees of participation in this process, with human cognition representing a high-complexity instantiation rather than a categorical departure. The framework further proposes that discontinuities in biological and cognitive complexity reflect threshold dynamics within a continuous field, rather than breaks in the underlying substrate.

The model remains incomplete. Key elements, including the formalization of the proposed operator grammar, the dimensional structure of the substrate, and the empirical detection of predicted thermodynamic signatures, require further development and experimental validation. Therefore, this study is offered not as a final account, but as a structured hypothesis: one that integrates existing findings across physics, neuroscience, and philosophy into a unified interpretive framework and that generates testable predictions regarding the relationship between information, topology, and conscious experience.

The framework does not close the question of consciousness. It reframes it.

## Data Availability

The datasets presented in this study can be found in online repositories.The datasets associated with this study are available in the Zenodo repository at: https://zenodo.org/records/19052006.
